# Exploring the Physical Properties of Cr_2_ZrP Full Heusler Alloy: A First Principles Study

**DOI:** 10.3390/ma19050882

**Published:** 2026-02-27

**Authors:** Wei Zheng, Chunmei Li, Yan Gao, Wenjiang Feng, Chuang Wu

**Affiliations:** 1 Basic Teaching and Research Department, Shenyang Urban Construction University, Shenyang 110167, China; 13840578890@163.com; 2 College of Physics Science and Technology, Shenyang Normal University, Shenyang 110034, China; cmli@synu.edu.cn (C.L.); gyan@synu.edu.cn (Y.G.); wjfeng@aliyun.com (W.F.)

**Keywords:** Cr_2_ZrP alloy, half-metallicity, first-principles calculation, spintronics

## Abstract

**Highlights:**

**What are the main findings?**

- Perfect half-metallicity and high spin polarization. Cr_2_ZrP exhibits 100% spin polarization at the Fermi level, with a metallic character in the minority spin channel and a clear band gap in the majority spin channel, making it an ideal candidate for spin injection.
- Robust magnetic properties and structural stability. The total magnetic moment of 3.00 μB follows the Slater-Pauling rule and remains perfectly integer even under a ±5% variation in lattice parameter, demonstrating exceptional resilience of its half-metallicity against lattice distortions.
- Excellent mechanical stability and ductile nature. The calculated elastic constants confirm mechanical stability. A high B/G ratio (~12.96) and Poisson’s ratio (~0.35) unequivocally predict a ductile behavior, which is crucial for practical device fabrication and mechanical reliability.

**What are the implications of the main findings?**

- The discovery of robust half-metallicity with 100% spin polarization in Cr_2_ZrP identifies them as promising candidates for next-generation spintronic applications.
- The combination of ferromagnetic behavior, structural stability, and mechanical ductility provides a strong theoretical foundation for the experimental design of high-performance spintronic materials.

**Abstract:**

As a new full Heusler compound, the Cr_2_ZrP alloy has attracted significant attention due to its potential applications in spintronics. In this paper, the electronic, magnetic, and mechanical properties of the Cr_2_ZrP alloy were systematically studied using first-principles calculations. The results show that the alloy is a half-metallic ferromagnet with high stability: it exhibits majority-spin-channel semiconductor behavior and minority-spin-channel metallic behavior at the Fermi level, with 100% spin polarization. The total magnetic moment is 3.00 μB, which is consistent with the Slater-Pauling behavior of half-metallic ferromagnets. When the lattice parameter changes by ±5%, the total magnetic moment and 100% spin polarization remain robust, demonstrating excellent mechanical magnetic coupling stability. The mechanical property analysis further revealed that Cr_2_ZrP meets the mechanical stability criterion of the cubic system and has a high bulk modulus (~172.8 GPa) and a high Debye temperature (~377 K). At the same time, its Pugh ratio (B/G ≈ 2.96) and Poisson ratio (ν ≈ 0.35) showed that the material had good ductility. The three-dimensional surface plot of Young’s modulus confirmed the obvious anisotropy of mechanical properties. This study theoretically confirmed that the Cr_2_ZrP alloy exhibits ideal half-metallic properties, robust magnetic order, good mechanical stability, and ductility, making it a promising candidate for future spintronic devices.

## 1. Introduction

Heusler alloys are intermetallic compounds with a specific stoichiometric ratio (X_2_YZ) and a highly ordered crystal structure. Since their discovery, owing to their highly tunable properties, diverse electronic structures, and exotic physical properties, these materials have become increasingly important in condensed matter physics and materials science. In recent years, with the rapid development of theoretical calculation and experimental techniques, research on Heusler alloys has made a series of breakthroughs, demonstrating significant application potential.

In theory, first-principles density functional theory calculations have become a powerful tool for the design and prediction of new Heusler alloys. By accurately calculating the energy band structure, researchers successfully predicted various Hessler compounds with topological properties. For example, Chadov et al. theoretically predicted that HgCr_2_Se_4_ and other materials are topological insulators, and the linear dispersion relationship near the Fermi level provides a new way to explore non-dissipative electron transmission [1]. Furthermore, some semi-magnetic Heusler alloys have been theoretically shown to be ideal platforms for realizing novel quantum states, such as axion insulators and weak semimetals. These discoveries have greatly expanded the scope of research on topological states [2,3].

In the field of experimental application, the breakthrough of the Heusler alloy is also remarkable. First, regarding the magnetic shape memory effect, Ni-Mn-Z (Z = Ga, In, Sn, Sb) and other systems exhibit up to 10% magnetic-field-induced strain, and their driving force arises from the strong coupling between the crystal structure and magnetic order during the martensitic transformation [4]. This feature shows excellent application prospects in MEMS actuators and efficient solid-state refrigeration technology (magnetocaloric effect). For example, the huge magnetic entropy change observed in the Ni-Mn-Sn alloy laid the foundation for the development of an environmentally friendly magnetic refrigerator [5]. Secondly, in the field of spintronics, all Heusler alloys, such as Co_2_MnSi and Co_2_FeAl, exhibit nearly 100% spin polarization and are ideal electrode materials for preparing high-performance magnetic tunnel junctions [6,7]. Experiments show that the magnetic tunnel junction with a Heusler alloy electrode can achieve an extremely high tunneling magnetoresistance, providing a key material system for the development of next-generation high-density, low-power nonvolatile magnetic random access memory [8]. In addition, Heusler alloys also show unique potential for thermoelectric energy conversion [9] and as a new type of magnetic Sigminzi carrier [10].

In summary, the collaborative breakthrough of theory and experiment has not only deepened our understanding of the fundamental physical problems of magnetic-electric coupling in Heusler alloys but also established its core position as a multifunctional quantum material and intelligent device. In this paper, the basic physical properties of the Heusler alloy Cr_2_ZrP will be studied using first-principles calculations. It is expected that this research will save experimental costs and provide an important theoretical basis for subsequent experimental research.

## 2. Materials and Methods

In this paper, the CASTEP module in Materials Studio 6.0 is used for first-principles calculations. The ultrasoft pseudopotential is used to describe the interaction between the real and valence electrons of the ion, and the exchange-correlation functional is the Perdew, Burke, and Ernzerhof form under the generalized gradient approximation. To ensure convergence and accurate calculation results, the plane-wave truncation energy is set to 500 eV. For the conventional cubic cell of the Heusler alloy, a 13 × 13 × 13 *k*-point grid generated by the Monkhorst–Pack method was employed to thoroughly sample the first Brillouin zone. A convergence test with respect to k-point sampling was performed, and the results confirm that the 13 × 13 × 13 grid ensures convergence of the total energy to within 1 meV/atom compared to a denser 15 × 15 × 15 mesh. This level of convergence is sufficient for accurately predicting the structural and energetic properties of the system. In the geometric structure optimization, the convergence criteria for the total energy, interatomic force, and internal stress are set to 2.0 × 10^−5^ eV/atom, 0.01 eV/Å, and 0.05 GPA, respectively. All calculations are performed under the condition of considering spin polarization. When calculating the band structure and magnetic properties of the Cr_2_ZrP alloy, the Coulomb interaction between d-orbital electrons of transition metal atoms is considered, and the GGA + U method is used, with U_Cr_ = 1.32 eV [11] and U_Zr_ = 1.40 eV.

## 3. Results and Discussion

### 3.1. Crystal Structure Stability

**Figure 1:**
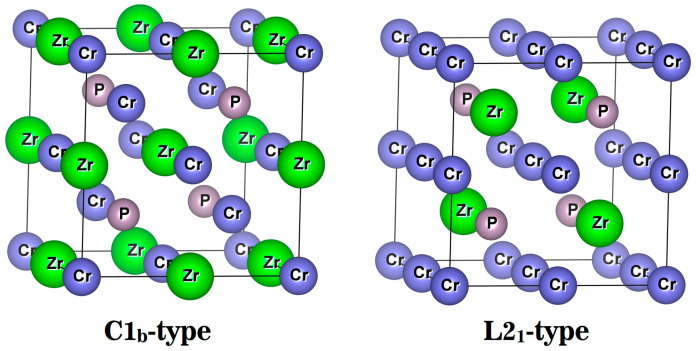
Two common competing phase structures in full Heusler alloy Cr_2_ZrP.

There are two common competing phase structures in the full Heusler alloy X_2_YZ: the L2_1_ phase (Cu_2_MnAl type, space group Fm-3m), which is the standard fully ordered Heusler alloy structure. The other is the C1_b_ phase (Hg_2_CuTi type, space group F-43m), an ordered half-Heusler alloy. The two phases of the Heusler alloy have a cubic structure and four independent Wyckoff positions, namely, A (0, 0, 0), B (0.5, 0.5, 0.5), C (0.25, 0.25, 0.25), and D (0.75, 0.75, 0.75). For the Cr_2_ZrP alloy, when two Cr atoms occupy positions A and B, it has an L2_1_ structure; when two Cr atoms occupy positions A and C, respectively, it forms a C1_b_ structure (as shown in Figure 1). Usually, for the transition metal atoms in full Heusler alloys, those with more valence electrons occupy the A and B positions first, whereas those with fewer valence electrons are more likely to occupy the C position. For the Cr_2_ZrP alloy, Cr has more valence electrons than Zr, so we predict that Cr_2_ZrP alloys are more likely to form Cu_2_MnAl-type alloys with L2_1_ structures.

**Figure 2:**
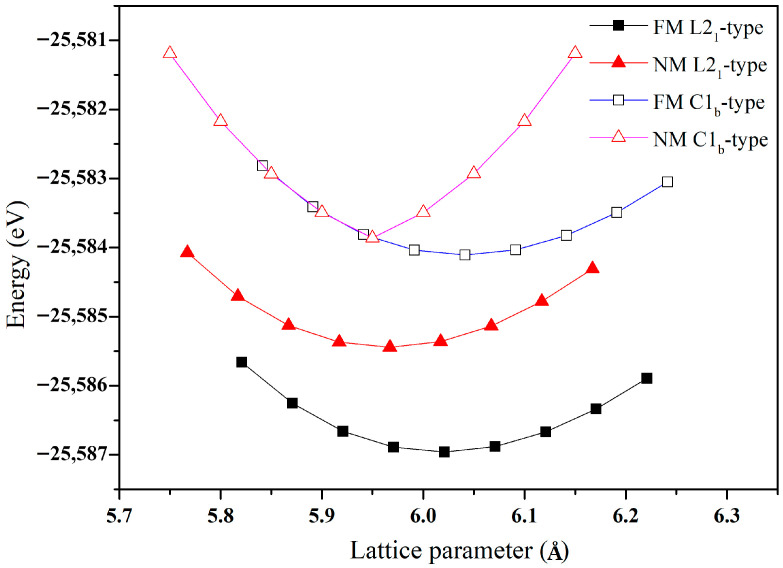
The energy to lattice parameter relation curve of the full Heusler alloy Cr_2_ZrP.

To determine the ground-state crystal structure of the Cr_2_ZrP alloy, we systematically considered two possible Heusler-type phases: the conventional cubic L2_1_ structure and the inverse Heusler (C1_b_) structure. For each phase, both ferromagnetic (FM) and non-magnetic (NM) configurations were considered. The total energy was calculated as a function of the lattice parameter for each configuration, and the resulting energy-volume curves are shown in Figure 2. Our calculations reveal that the L2_1_-type Heusler structure in the FM state exhibits the lowest total energy, with an optimized equilibrium lattice constant of a_0_ = 6.02 Å, indicating that this configuration represents the most stable ground state for Cr_2_ZrP.

To date, no experimental or theoretical data for the Cr_2_ZrP alloy have been reported in the literature. Therefore, the structural parameters and magnetic ground state presented here serve as predictions for this compound and are expected to provide useful guidance for future experimental synthesis and characterization. To further contextualize our findings, we compared the properties of Cr_2_ZrP with those of the related alloys Cr_2_ZrSi [11] and Fe_2_ZrP [12,13], which share similar structural characteristics.

In addition, in the development of new alloy materials, the formation energy is the key thermodynamic parameter to measure the stability of a compound relative to its constituent elements in the free state. The negative formation energy indicates that the compound is thermodynamically possible to form, and the smaller the value, the more stable the structure is. The formula for calculating the formation energy of the Cr_2_ZrP alloy is given [14]:

(1) $$\Delta E=E_{Cr_{2}ZrP}-\left(2E_{Cr}+E_{Zr}+E_{P}\right).$$

Here, *E*_Cr2ZrP_ is the total energy of the optimized Cr_2_ZrP cell, and *E*_Cr_, *E*_Zr_, and *E*_P_ are the chemical potentials of Cr, Zr, and P atoms, respectively. The calculated formation energy of the Cr_2_ZrP alloy is −3.831 eV (Table 1), which is smaller than the formation energy of the Cr_2_ZrSi alloy, indicating that the Cr_2_ZrP alloy has better thermodynamic stability. However, thermodynamic stability alone does not guarantee dynamic stability. To fully validate the structural stability of Cr_2_ZrP, phonon dispersion calculations are required to confirm the absence of imaginary frequencies. Although such calculations were not performed in the present work due to computational resource limitations, they represent a crucial step in our ongoing investigation and will be addressed in future studies.

### 3.2. Magnetic and Half-Metallic Properties

In the study of spintronic materials, half-metallic ferromagnets have attracted much attention due to their unique electronic structure. These materials are metallic in one spin channel and semiconductor in the other, resulting in 100% spin polarization. This characteristic is an ideal choice for achieving high-efficiency spin injection, which is very important for the development of next-generation spintronic devices (such as magnetic random access memory and spin field-effect transistors). Previous studies have shown that alloys with Heusler or anti-Heusler structures are important carriers of semimetallic properties. On the basis of confirming the structural stability of the Cr_2_ZrP alloy, the band structure and density of states of the Cr_2_ZrP alloy were systematically analyzed by first-principles calculation to reveal its magnetic properties and explore whether it has semimetallic properties.

**Figure 3:**
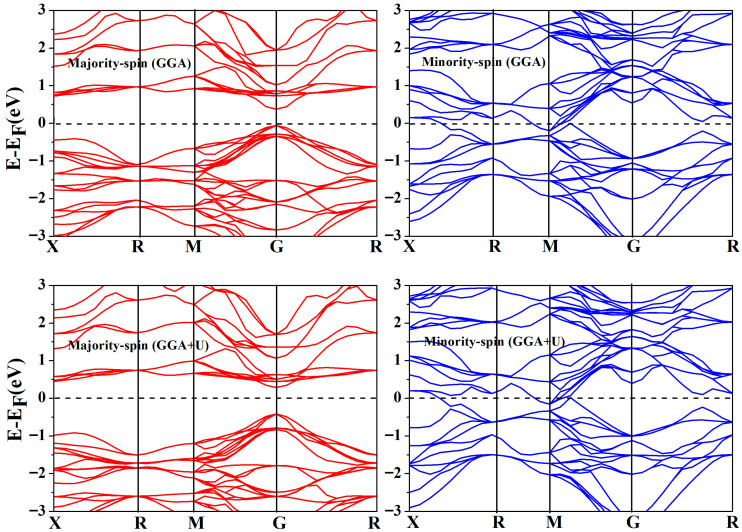
The energy band structure of Cr_2_ZrP alloy, calculated using the approximate methods of GGA and GGA + U.

As shown in Figure 3, the energy band structure of the Cr_2_ZrP alloy was calculated using the approximate GGA and GGA + U methods. The calculation results show a noticeable band gap in the energy bands of the majority-spin channels at the Fermi level, indicating that electrons in the spin direction cannot be excited to participate in conduction, thereby demonstrating semiconductor characteristics. In minority-spin channels, the Fermi energy levels cross multiple energy bands, indicating that the channel is metallic and that there are many free carriers. This energy band characteristic, in which one spin channel is a metal and the other spin channel is an insulator, is a typical symbol of half-metallic materials. Compared with the band structures of majority-spin channels, when considering Coulomb potential interactions between transition metal elements (GGA + U), the Fermi level shifts, resulting in a larger energy band gap between the conduction and valence bands.

In spintronics, spin polarizability is a key parameter for evaluating the degree of spin polarization of materials at the Fermi level, which directly affects spin injection efficiency, magnetoresistance, and the performance of spin devices [15]. High-spin polarizability materials, such as half-metallic ferromagnets, can achieve nearly 100% spin polarization because only one spin-oriented electronic state dominates at the Fermi level, whereas the other spin channel has an energy gap [16]. Spin polarizability P is usually defined as the ratio of the difference between the spin up and spin down density of states at the Fermi level and the total density of states. The formula is as follows [17]:

(2) $$P=\frac{N_{↑}\left(E_{F}\right)-N_{↓}\left(E_{F}\right)}{N_{↑}\left(E_{F}\right)+N_{↓}\left(E_{F}\right)},$$

where and represent the spin up and spin down electron density of states at the Fermi level, respectively. For the Cr_2_ZrP alloy, *P* = 1, corresponding to 100% spin polarizability. This shows that the Cr_2_ZrP alloy allows only spin-up electrons to participate in transport at the Fermi level, making it a potential material for spintronic applications.

**Figure 4:**
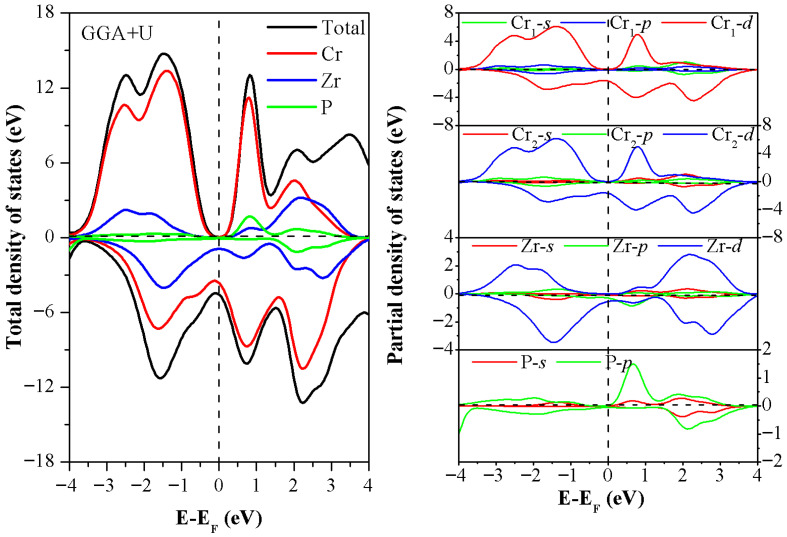
The total density of states and partial density of states of Cr_2_ZrP alloy.

The Cr_2_ZrP alloy is a promising new half-metallic ferromagnetic material. Its macromagnetism is rooted in its microelectronic structure. The total density of states (TDOS) and partial density of states (PDOS) obtained by first-principles calculations provide a key perspective for understanding the magnetic mechanism, especially the roles of atoms in forming magnetic moments and generating band gaps. The analysis of the total density of states of the Cr_2_ZrP alloy (Figure 4) shows a significant band gap in the majority-spin channel, whereas in the minority-spin channel, the Fermi level passes through a finite density of states, indicating metal-like characteristics. This asymmetric electronic structure is the hallmark of a typical half-metallic ferromagnet.

In order to reveal the source of the magnetism of the Cr_2_ZrP alloy, it is necessary to analyze the partial density of states of each constituent atom (Figure 4). We found that the 3d electron of the Cr atom is the primary contributor to the magnetism of the Cr_2_ZrP alloy. Its PDOS shows strong asymmetry in the majority-spin and minority-spin channels. In the vicinity of the Fermi level, minority-spin d-electron states dominate, whereas majority-spin d-electron states are strongly suppressed at the Fermi level, and a clear band gap is formed. This significant difference in the density of states directly leads to each Cr atom carrying a significant local magnetic moment, which is the primary source of the alloy’s total magnetic moment [18]. The contribution of the 4d electrons of the Zr atom to the total magnetic moment is small, but its role cannot be ignored. There is some hybridization between the d orbitals of Zr and Cr. This hybrid effect not only slightly induces a small polarized magnetic moment of the Zr atom itself (usually antiparallel to the magnetic moment of the Cr atom, i.e., antiferromagnetic coupling) but also helps stabilize the crystal field environment in the majority-spin channel and plays a key regulatory role in opening the band gap in the Cr-d energy band [19]. The density of states of 3p electrons of the P atom near the Fermi level is very low. Therefore, the direct contribution of the P atom to the total magnetic moment is minimal. However, as an interstitial atom, P acts as a “bridge” in the crystal structure. Its p orbital hybridizes with the d orbital of transition metal atoms (Cr, Zr), mediating the exchange interaction between atoms, helping establish long-range magnetic order and solidifying the spin-polarised energy band structure [20].

In summary, the magnetism of the Cr_2_ZrP alloy is the result of the cooperative action of various constituent atoms: the 3d electrons of Cr provide the main body of the magnetic moment and the basis of half-metallicity. The 4d electrons of Zr participate in and stabilize the formation of the band gap through orbital hybridization. The 3p electrons of P maintain the overall ferromagnetic order by mediating exchange interactions. This microscopic analysis based on the density of states provides a solid theoretical basis for understanding and designing high-performance semimetallic spin injection materials.

**Figure 5:**
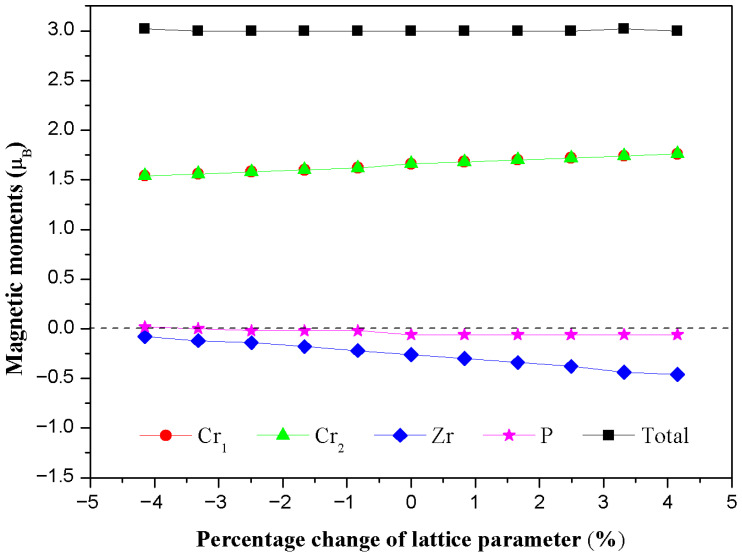
The relationship curve between the total magnetic moment and the atomic magnetic moment of Cr_2_ZrP alloy and the lattice parameter.

In the design of spintronic devices, the magnetic and structural stability of half-metallic ferromagnets is crucial. Epitaxial stress or thermal fluctuations during the actual preparation process may cause the lattice parameter to deviate from its theoretical equilibrium value. Therefore, exploring the relationship between the magnetic properties of materials and their lattice parameters is a key link for evaluating their practical application potential [21]. In order to study the response behavior of the total magnetic moment and the atomic magnetic moment of the Cr_2_ZrP alloy under lattice tension or compression, we calculated the relationship between the total magnetic moment and the atomic magnetic moment of the Cr_2_ZrP alloy and the lattice parameter (Figure 5). The calculation results reveal a key phenomenon: when the lattice parameter a changes from −5% to +5%, the total magnetic moment of the alloy is always stable at an integer value of 3.00 μB. This result strictly follows the Slater Pauling behavior of the half-metallic ferromagnet and shows that its 100% spin polarizability is ideally maintained in this lattice interval [22].

Further analysis of the atomic magnetic moments reveals that the local magnetic moment of each Cr atom increases slightly upon lattice expansion. As the primary contributor to total magnetization, this increase can be attributed to the enhanced localization of Cr-3d electrons resulting from reduced orbital overlap at larger interatomic distances. Notably, the variation in the absolute magnetic moment of Cr remains below 5% across the considered lattice range, indicating strong intrinsic magnetic stability. The Zr atom carries a small induced magnetic moment that is antiparallel to that of the Cr atoms. Consequently, despite the formal assignment of a ferromagnetic (FM) ground state based on total energy comparisons, the magnetic ordering in Cr_2_ZrP more accurately corresponds to a ferrimagnetic configuration, characterized by unequal opposing moments. This distinction is physically significant, as the net magnetic moment arises from the partial cancelation between the dominant Cr and weaker Zr sublattices. In contrast, the magnetic moment induced on the P atom remains negligibly small (close to zero) throughout the lattice deformation and exhibits no detectable sensitivity to changes in the lattice parameter. This behavior is consistent with the non-magnetic nature of P in this compound, where its valence electrons are primarily involved in covalent bonding with the transition metal atoms rather than contributing to the magnetic ordering. The total magnetic moment remains remarkably stable against lattice variation, which we attribute to the robust hybridization and effective antiferromagnetic coupling between Cr atoms via P-mediated indirect exchange interactions.

### 3.3. Mechanical Properties

New spintronic materials need not only excellent magnetoelectric properties but also good structural stability and mechanical properties to withstand various stress environments in practical applications. As a potential semimetallic ferromagnet, the evaluation of the mechanical properties of the Cr_2_ZrP alloy is critical. based on first-principles calculations, the mechanical stability, ductile-brittle transition, anisotropy, and Debye temperature of Cr_2_ZrP are systematically analyzed by calculating its elastic constants at the ground state, which provides a theoretical basis for its feasibility in device manufacturing.

First, for a cubic Heusler alloy, there are three independent elastic constants: *C*_11_, *C*_12_, and *C*_44_. Elastic constants can judge the mechanical stability of the material. The traditional mechanical stability criterion is [23]:

(3) $$\left\{\begin{array}{c} C_{11}>0\begin{array}{cc} , & C_{44}>0 \end{array}\\ C_{11}>\left|C_{12}\right|\\ C_{11}+2C_{12}>0 \end{array}\right..$$

The values of various physical quantities characterizing the mechanical properties of the Cr_2_ZrP alloy, as calculated by us, are shown in Table 2. The calculation results show that the Cr_2_ZrP alloy meets the mechanical stability criteria.

Second, by calculating the Pugh’s ratio and Poisson’s ratio of the system, the ductility and brittleness of the material can be characterized. Generally, there are two critical values to distinguish ductile materials from brittle materials, namely B/G = 1.75 and ν = 0.25 [24]. Materials above this critical value have ductile behavior. The physical quantities characterizing the above mechanical properties can be obtained from the following formula:

(4) $$B=\frac{C_{11}+2C_{12}}{3},$$

(5) $$G=\frac{C_{11}-C_{12}+3C_{44}}{5},$$

(6) $$\nu =\frac{3B-2G}{2\left(3B+G\right)}.$$

The calculated results are shown in Table 2. The high B/G ratio and Poisson’s ratio indicate that Cr_2_ZrP exhibits significant ductile tendency, which is essential for resisting cracking during processing into thin films or bonding to substrates. However, the calculated bulk modulus-to-shear modulus ratio (B/G) of approximately 2.96 indicates a ductile tendency, as per the Pugh criterion (B/G > 1.75). Although this value is relatively high compared to most Heusler alloys, it aligns with the trend reported in related Cr-based compounds such as Cr_2_ZrSi. Given the unusually large magnitude of this ratio, experimental verification of the mechanical properties would be particularly valuable.

Third, to evaluate the direction dependence of mechanical properties, we calculated the anisotropy index of Young’s modulus. In the cubic crystal system, the formula of the anisotropy constant is as follows [25]:

(7) $$A=\frac{2C_{44}}{C_{11}-C_{12}}.$$

**Figure 6:**
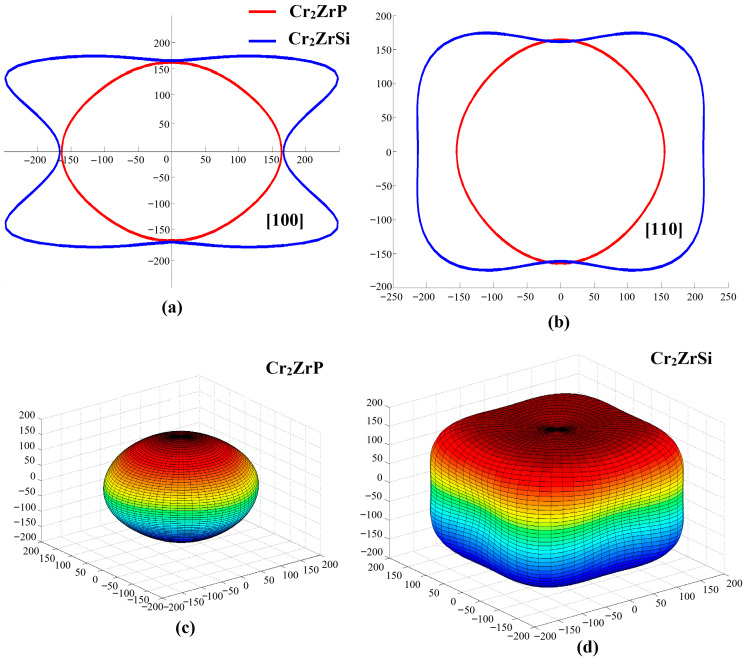
The Young’s modulus distributions of Cr_2_ZrP and Cr_2_ZrSi alloys in various directions: (**a**,**b**) represent the Young’s modulus distribution of two alloys on the [100] and [110] surfaces, respectively; (**c**,**d**) represent the 3D Young’s modulus surface distribution for two alloys, respectively.

Calculated *A* = 0.92. If *A* = 1, it is completely isotropic; When *A* ≠ 1, it shows anisotropy. The value of Cr_2_ZrP deviates significantly from 1, indicating that its Young’s modulus has clear anisotropy. In addition, the degree of anisotropy can also be determined by calculating Young’s modulus distribution in all directions of the crystal material, which satisfies the following relationship [26]:

(8) $$\frac{1}{E}=S_{11}-2\left(S_{11}-S_{12}-\frac{S_{44}}{2}\right)\left(l_{1}^{2}l_{2}^{2}+l_{2}^{2}l_{3}^{2}+l_{3}^{2}l_{1}^{2}\right),$$

where *S*_11_, *S*_12_, and *S*_44_ are the inverse matrices of the elastic constants *C*_11_, *C*_12_, and *C*_44_, respectively, and *l*_1_, *l*_2_, and *l*_3_ are the directional cosines. Using the above formula, the Young’s modulus distribution of Cr_2_ZrP and Cr_2_ZrSi alloys in all directions is shown in Figure 6. The distribution diagram shows the anisotropic characteristics of Young’s modulus of Cr_2_ZrP intuitively. We find that Cr_2_ZrP exhibits only weak elastic anisotropy, with the Young’s modulus varying within a narrow range across different crystallographic directions. The three-dimensional surface representation is nearly spherical, further confirming that the mechanical properties of Cr_2_ZrP tend toward isotropy. Compared with the Cr_2_ZrSi alloy, the anisotropy of the Cr_2_ZrP alloy is weakened. This near-isotropic behavior is advantageous for applications requiring uniform stress distribution.

Finally, the Debye temperature *θ*_D_ is the key physical quantity connecting the elastic and thermal properties of materials. We use the average sound velocity model to derive the longitudinal wave sound velocity v*_l_* and the transverse wave sound velocity v*_s_* from the bulk elastic modulus and shear modulus and then calculate the Debye temperature of Cr_2_ZrP. The relevant physical quantity formula is as follows [27,28]:

(9) $$v_{s}={\left(\frac{G}{\rho }\right)}^{\frac{1}{2}}{\begin{array}{cc} , & v_{l}=\left(\frac{3B+4G}{3\rho }\right) \end{array}}^{\frac{1}{2}},$$

(10) $$v_{m}={\left[\frac{1}{3}\left(\frac{2}{v_{s}^{3}}+\frac{1}{v_{l}^{3}}\right)\right]}^{-\frac{1}{3}},$$

(11) $$\theta_{D}=\frac{h}{k_{B}}{\left[\frac{3n}{4\pi V_{a}}\right]}^{\frac{1}{3}}v_{m}.$$

The calculated Debye temperature of Cr_2_ZrP is about 377 K. The higher Debye temperature indicates that the alloy has strong interatomic bonding, good thermal stability, and high thermal conductivity, which are conducive to rapid heat dissipation during the operation of spintronic devices and to the avoidance of performance degradation caused by heat accumulation. It should be emphasized that this estimation does not account for detailed phonon dispersion or phonon-phonon scattering effects. Consequently, the computed value functions as a fundamental material parameter for comparative analysis with related compounds, rather than as a quantitative prediction of thermal conductivity. Future experimental measurements or explicit phonon transport calculations would be essential to validate this preliminary estimation.

In summary, the elastic constant calculation shows that the Cr_2_ZrP alloy is not only mechanically stable but also has good ductility, moderate mechanical anisotropy, and a high Debye temperature. These excellent, comprehensive mechanical properties, combined with the previously reported half-metallic properties, make it an attractive candidate for future spin-electronic device applications.

## 4. Conclusions

In this study, the physical properties of the Cr_2_ZrP alloy were comprehensively evaluated using first-principle calculations. The main conclusions are as follows:

First, half-metallic properties and electronic structure: Cr_2_ZrP exhibits a typical semimetallic electronic structure at the Fermi level. The majority spin channel has a clear energy gap, whereas the minority spin channel is in a metallic state, which achieves 100% spin polarizability. This characteristic enables it to serve as an efficient spin-injection source, an ideal basis for the application of spin valves, magnetic tunnel junctions, and other core spin-electronic components.

Second, magnetic properties and stability: The total magnetic moment of the alloy is 3.00 μB, and it is mainly contributed by 3d electrons of Cr atoms. When the lattice parameter changes significantly (±5%), the total magnetic moment always maintains an integer quantum number, and the half-metallic properties are not destroyed. This robust magnetism, which is insensitive to lattice distortion, dramatically enhances the reliability of the material in actual device fabrication and operating environments, where substrate mismatch or thermal stress may be present.

Third, mechanical properties and stability: The calculation of elastic constants confirms that Cr_2_ZrP is mechanically stable. Its high bulk modulus and Debye temperature indicate that the material has high hardness and thermal stability. At the same time, the ductility analysis based on the Pugh criterion and Poisson’s ratio indicates that the material is ductile, helping to avoid brittle fracture during processing and use and improving machining feasibility.

Fourth, comprehensive application prospects: In summary, the Cr_2_ZrP alloy successfully combines its inherent half-metallic magnetism (functionality) with good mechanical stability and ductility (structure). This comprehensive performance advantage not only gives it excellent spin-transport characteristics but also the potential to withstand various mechanical stresses during actual device manufacturing.

This study laid a foundation for the theoretical exploration of Cr_2_ZrP. Future work should focus on the experimental synthesis and verification of the material, as well as on further studies of its film morphology, bonding quality with common substrates, and specific performance in nanoscale devices.

It should be emphasized that the current computational framework does not incorporate spin–orbit coupling (SOC) effects. Although SOC is anticipated to be relatively weak for Cr-3d electrons, its influence on Zr-4d states may prove non-negligible, potentially inducing minor modifications to the band structure proximate to the Fermi level. Nevertheless, due to the substantial computational resources required for SOC calculations in this system, such an investigation exceeded the scope of this study. Subsequent research integrating SOC would be essential to unequivocally validate the robustness of the predicted half-metallicity in Cr_2_ZrP.

## Figures and Tables

**Table 1:** The equilibrium lattice parameter, total energy, formation energy, and magnetic moments of the full Heusler alloy Cr_2_ZrP.

		Cr_2_ZrP	Cr_2_ZrSi [11]	Fe_2_ZrP [12]
Lattice parameter/Å		6.02	6.160	5.870
formation energy/eV		−3.831	−0.150	-
Magnetic moments/$\mu_{B}$	Cr_1_	1.66	-	-
	Cr_2_	1.66	-	-
	Zr	−0.28	-	-
	P	−0.04	-	-
	Total	3	2	1

**Table 2:** Elastic constants, Bulk modulus, Shear modulus, Young modulus, Anisotropy ratio, Poisson’s ratio, Pugh’s ratio, Average sound velocities, and Debye temperatures of full Heusler alloy Cr_2_ZrP.

		Cr_2_ZrP	Cr_2_ZrSi [11]
Elastic constants	C_11_ (GPa)	254.5	238.589
	C_12_ (GPa)	131.9	117.022
	C_44_ (GPa)	56.3	96.028
Bulk modulus	B (GPa)	172.8	157.544
Shear modulus	G (GPa)	58.3	79.940
Pugh’s ratio	B/G	2.96	2.731
Poisson’s ratio	*ν*	0.35	0.337
Anisotropy constant	A	0.92	1.580
Longitudinal wave sound velocity	*v_l_*	6050.595	6450.774
Transverse wave sound velocity	*v_s_*	2918.904	2548.822
Average sound velocity	*v_m_*	3281.104	3955.533
Debye temperature	*θ*_D_	377.196	303.848

## Data Availability

The original contributions presented in this study are included in the article. Further inquiries can be directed to the corresponding author.
